# Ruminant livestock and climate change: critical discourse moments in mainstream and farming sector news media

**DOI:** 10.1007/s10460-024-10651-7

**Published:** 2024-11-11

**Authors:** Philippa Simmonds, Damian Maye, Julie Ingram

**Affiliations:** Countryside and Community Research Institute, University of Gloucestershire, Francis Close Hall Campus, Swindon Road, Cheltenham, Gloucestershire, GL50 4AZ UK

**Keywords:** Ruminant livestock, Climate change, Media analysis, Critical discourse moments, Discursive power, Polarisation

## Abstract

There is ongoing contestation around greenhouse gas emissions from ruminant livestock and how society should respond. Media discourses play a key role in agenda setting for the general public and policymakers, and may contribute to polarisation. This paper examines how UK news media portrayed ruminant livestock’s impact on climate change between 2016 and 2021. The analysis addresses a gap in the literature by comparing discourses in national and farming sector newspapers using a qualitative approach. Four national and two farming sector news outlets were searched for articles published between 2016 and 2021. A corpus of 996 relevant articles was assembled, from which 154 were selected for in-depth examination using Critical Discourse Analysis. Four ‘Critical Discourse Moments’ (CDMs), each signifying a discursive shift in the debate, were identified over the 6-year studied period: 1) Low salience, diverging discourses, 2) We must eat far less meat, 3) Fighting the anti-meat agenda, and 4) Policy (in)action at COP26. There was a large increase in the number of published articles from January 2019 onward, partly associated with publication of the EAT/Lancet Commission report. CDM 2 (We must eat far less meat) occurred mainly in the national media, while CDM 3 (Fighting the anti-meat agenda) occurred mainly in the farming media. Our findings reveal both opinion polarisation and intergroup polarisation between national and farming sector media, and low engagement with food system power imbalances. Addressing polarisation will be important to enhance capacity for collective decision-making regarding methane emissions from ruminant livestock.

**Supplementary Information:**

The online version contains supplementary material available at 10.1007/s10460-024-10651-7.

## Introduction

This paper examines how UK news media portrayed ruminant livestock’s impact on climate change between 2016 and 2021. Ruminant livestock, specifically cows and sheep, emit methane as a by-product of their biological approach to digesting cellulose. Along with carbon dioxide and nitrous oxide, methane is a greenhouse gas (GHG) that must be limited if countries are to achieve their commitments under the Paris Agreement. Globally, approximately one third of anthropogenic methane emissions arise from ruminant enteric fermentation and manure management (Saunois et al. 2020). However, addressing ruminant methane emissions is often contentious, exposing a range of debates spanning land use, human diet and health, and the role of the state (Simmonds and Vallgårda 2021).

Ruminant production is, of course, linked with red meat consumption. The quantity of meat consumed globally continues to rise, with low- and middle-income countries seeing increasing consumption as they develop (Hansen and Jakobsen 2020), and relatively rapid increases in consumption of pig and poultry meat (Lundström 2019). Populations of high-income countries such as the UK and the USA tend to consume more meat than global averages, with red meat consumption decreasing yet remaining prevalent (Stewart et al. 2021). Recent years have seen numerous high profile reports calling for reductions in meat production and consumption; particularly red meat consumption in high income ‘Western’ countries including the UK and the USA (Shukla et al. 2019; Willett et al. 2019; Climate Change Committee 2020a). However, these messages are often contested by domestic livestock industries (Lazarus et al. 2021; Larsson and Vik 2023).

This paper focuses specifically on UK news media. The UK has many regions specialised in ruminant livestock production (DEFRA 2023), and a diet that features high meat consumption compared to national nutrition guidelines (Stewart et al. 2021). The UK has achieved significant overall emission reductions, mainly through the energy sector, while agricultural emissions have remained broadly static over the past decade (Ward 2023). There is a growing awareness that achieving the legislative goal of net zero by 2050 will require changes that impact daily lives, including shifts in diet (Climate Change Committee 2023). There is also increasing focus on the farming sector for its potential to both reduce emissions and increase carbon sequestration as part of a net zero economy (Climate Change Committee 2020b). This situation has inflamed multiple polarised debates around ruminant livestock’s future role in the UK’s food system, reflecting global concerns and those particular to high-income industrialised countries (Farstad et al. 2020). Meanwhile, UK newspapers have significant international readership and therefore may influence media discourse in other countries (Kristiansen et al. 2020).

Public attitudes and culture are recognised as barriers to achieving sustainable transformations in food systems (Conti et al. 2021). The media plays a key role in this regard; creating, reproducing, and contesting societal meanings (Burgess 1990; Corbett and Durfee 2004; Carvalho 2008). Despite increasing prominence of social media for sharing news, traditional news media outlets still play a significant role in agenda setting (Carvalho 2007; Friedlander et al. 2014; Happer and Wellesley 2019), and can be used as a tool of persuasion by various actors (Donnison et al. 2023). There is likely a bidirectional influence between news media and the general public, with both contributing to issue framing and agenda setting (Zhou and Moy 2007; Ragas et al. 2014). If an issue is discussed more prominently in the media, it is said to be more salient, and this contributes to agenda setting amongst readers (Scheufele and Tewksbury 2007); frequency of articles is one way to measure issue salience (Rust et al. 2021).

In high income countries there has been a historic increase in the proportion of newspaper articles on climate change that mention food system contributions, as well as those mentioning animal agriculture specifically (Neff et al. 2009; Almiron and Zoppeddu 2015; Mroz and Painter 2023). A related body of scholarship within Critical Animal Studies (CAS) has critiqued the “cultural hegemony of meat” (Fitzgerald and Taylor 2014) within news media. This approach shows how discourses, including within advertisements and news articles, normalise and reinforce the superiority of humans and the commodification of (certain) animals to consumer audiences (Fitzgerald and Taylor 2014; Cole 2016; Khazaal and Almiron 2016). Despite this literature, evidence suggests that in recent years, UK national newspapers may have been contributing to a process of “de-meatification” by supporting “less meat initiatives” and advocating reduced or even no meat consumption (Morris 2018; Mroz and Painter 2023). Prior research has shown that news media usually highlights individual consumer dietary choices as the locus of concern in debates around ruminant livestock and climate (Kristiansen et al. 2020; Mroz and Painter 2023), as well as in debates about red meat and human health (Wells 2017).

The increased salience of meat in relation to climate has also been seen on social media. Conversations around meat are prevalent on Twitter (Maye et al. 2021), and Sanford et al. (2021) demonstrated a high level of toxicity among Twitter responses to the IPCC’s Special Report on Climate Change and Land (Shukla et al. 2019). The authors suggest this shows polarisation on the topic of meat and climate (Sanford et al. 2021). Polarisation can inhibit environmental decision-making and hinder efforts to act on climate change (Judge et al. 2023). For example, research on Swedish media constructions of farmers as heroes or villains before and after the publication of the FAO’s Livestock’s Long Shadow report (Steinfeld et al. 2006) suggests that these polarised identities may inhibit motivation for farmers to engage in pro-environmental activity, or may even contribute to them leaving farming altogether (Hallgren et al. 2020).

Agri-food scholars have previously explored key facets of debates around ruminant livestock and climate, such as narratives around alternative proteins (Sexton et al. 2019), livestock sector engagement with GHG metrics (Lynch et al. 2020; Cusworth et al. 2023), and sustainable agriculture narratives (Bless et al. 2023). This research speaks to barriers to sustainable food system transformation, specifically those pertaining to attitudes and cultures (Conti et al. 2021). Previous research into media representations of the relationship between ruminant livestock and climate change has tended to favour quantitative approaches (Neff et al. 2009; Friedlander et al. 2014; Almiron and Zoppeddu 2015; Kristiansen et al. 2020; Mroz and Painter 2023), and a gap therefore exists in terms of using qualitative analysis with a more explicit focus on power. To address this gap, the rest of this paper is structured as follows. The next section discusses major trends in public climate discourse, and proposes Critical Discourse Analysis as a conceptual tool to identify and describe major discourses and power dynamics pertaining to ruminant livestock and climate. The Methodology section describes how articles were searched for and selected for inclusion, including a focused sub-set of articles for in-depth analysis. The findings are then presented, organised as four Critical Discourse Moments. The final section discusses the findings and their international implications, relating them to the need to address (perceived) polarisation to mitigate climate delay.

## Understanding news media discourse, including the use of “critical discourse moments”

### Recent trends in public climate change discourse

In recent years, climate scholars have identified a turn away from outright climate change denial towards “discourses of climate delay” in news media, policy documents, and political rhetoric (Lamb et al. 2020). These are discursive strategies that seek to delay action on climate change, shown in Table 1. For example, “Whataboutism” refers to the tendency to deflect attention towards other sectors or countries, emphasising their contributions to global emissions. These discourses build on legitimate concerns about rapid climate action, but move into misrepresentation and imply that change is neither desirable nor possible (Lamb et al. 2020).

**Table 1:** The discourses of Climate Delay, adapted from Lamb et al. (2020)

Category	Discourse	Example
Redirect responsibility	Whataboutism	Our carbon footprint is trivial compared to […] Therefore it makes no sense for us to act, at least until […] does.
	Individualism	Individuals and consumers are responsible for solving climate change.
	The “free rider” excuse	Reducing emissions would weaken us, and others would take advantage of that.
Push non-transformative solutions	Technological optimism	We should focus on current and future technologies.
	All talk, little action	We have declared a climate emergency and set an ambitious target.
	Fossil fuel solutionism	Fossil fuels are becoming more efficient and are the bridge towards a low-carbon future.
	No sticks, just carrots	Society will only respond to incentives; restrictive measures will fail and should be abandoned.
Emphasise the downsides	Policy perfectionism	We should seek perfectly crafted solutions that are supported by all affected parties.
	Appeal to well-being	Fossil fuels are essential for development, thus abandoning them will deny the global poor their right to modern lifestyles.
	Appeal to social justice	Climate action will be expensive, and the vulnerable members of our society will bear the burden. Hard working people cannot enjoy their holidays.
Surrender	Change is impossible	Any measure to significantly reduce emissions is counter to human nature and therefore impossible to implement in a democratic society.
	Doomism	Catastrophic climate change is already locked in, we should just accept our fate.

Meanwhile, a particular challenge to political action on methane emissions from ruminant livestock is that the arena is often considered to be polarised (Sanford et al. 2021). Polarisation describes “a situation in which strongly held opposing opinions form around an issue in society, creating a sense of ‘us versus them’ divides” (Judge et al. 2023, p. 1). Media discourse forms part of people’s personal information environments, and thus may influence opinion (Happer and Wellesley 2019). Moreover, polarisation may be actual or perceived, and scholars have differentiated between “opinion polarisation” and “intergroup polarisation” (Judge et al. 2023). Opinion polarisation indicates a bimodal clustering of opinions, with a significant lack of agreement between the two groups. Intergroup polarisation is about group members’ perceptions of the opposing group, and their perceptions of the opposing group’s opinions. If there is a significant level of negative emotion directed toward a perceived outgroup, then this is intergroup polarisation. Intergroup polarisation can be increased by animosity and negativity (including in news media) between opposing groups (Judge et al. 2023). However, research has shown that actual polarisation is lower than perceived polarisation; people think that society is more polarised than it really is (Lees and Cikara 2021; Sparkman et al. 2022; Judge et al. 2023). While groups with extreme views at either end of the opinion spectrum may be polarised, most people are exposed to heterogenous opinions and have more moderate views. Both actual and perceived polarisation are problematic for environmental decision-making, as they discourage conversations around climate and reduce capacity for collective action (Judge et al. 2023).

### Critical discourse analysis and critical discourse moments

Given the influence of media on social values and opinions, and the propensity of actual and perceived polarisation to discourage collective dialogue on climate change, it is essential to undertake analyses to better understand media discourses around contentious topics. This paper uses Critical Discourse Analysis (CDA) as an interpretive tool. CDA typically aims to explicitly examine discursive power, defined as the power to influence the norms and values that guide behaviour (Sievert et al. 2020). CDA emerged in the later 20th century and draws on neo-Marxist ideas about class and power relations. Analytical emphasis is on language in the creation, framing, and understanding of social interactions and social problems (Fairclough 1992; Bischoping and Gazso 2016). CDA is broadly pluralist, in that it features certain ontological contradictions. On one hand, it takes the constructionist perspective that discourse is socially constructed and constituted by power-knowledge relations. On the other, it adopts the activist standpoint of realist critical theory, committed to emancipation and social change in the real world (Maeseele 2015). This means that CDA considers discourses to have both social and material drivers and consequences (Bischoping and Gazso 2016). A key critique of CDA is that the researcher’s interpretation of a text does not necessarily correspond with the average reader’s interpretation, thus one can only speculate as to its real-world impacts (Blommaert and Bulcaen 2000). This critique can be partially addressed via contextualisation (Carvalho 2008).

Scholars have previously used CDA to better understand issues related to farming and climate change. A 2015 study demonstrated how media discourse around genetically modified crops facilitated or impeded democratic debate by contributing to processes of politicisation and de-politicisation (Maeseele 2015). Previous work on climate change discourse in UK media showed how ideological standpoints of different newspapers had impacts on various dimensions of science communication, including the interpretation of “facts”, the recognised agents of definition, and the goals associated with knowledge (Carvalho 2007). Another study used CDA to identify climate change metaphors used in a Swedish farming magazine (Asplund 2011). Three metaphors were identified, each emphasising specific aspects of climate change without portraying the whole picture, and tending to explicitly or implicitly advocate climate change mitigation, rather than adaptation.

This paper adopts the methodological approach proposed by Carvalho (2008), which was developed specifically for news media and has been applied in the UK. The approach employs a timeline perspective, focusing on events and periods that caused discourses to shift and re-form. These are known as “critical discourse moments” (Carvalho 2008). Critical discourse moments (CDMs) are events that “make discourse on an issue especially visible” (Gamson 1992, p.26). They provoke stakeholders to reassert discourses and use these to interpret the latest development. Carvalho (2008) defines CDMs as:

…Periods that involve specific happenings, which may challenge the “established” discursive positions. Various factors may define these key moments: political activity, scientific findings or other socially relevant events. (Carvalho 2008, p.166)

She argues that as time passes, discourses calcify and become more recurrent. Researchers have previously used CDMs to analyse coverage of the early 2000s SARS epidemic in Belgian media, identifying four “moments” in the corpus, which comprised 57 news items (Joye 2010). Using a CDM approach allows researchers to show how discourses evolve over time and in response to contextual events, rather than simply providing a “snapshot” at one point in time. This is valuable for identifying patterns and improving predictions of responses to future events.

### National news media sources

In terms of national news media, previous research on climate and ruminant livestock has selected “elite media” to analyse, due to the levels of trust and readership these publications enjoy, and their orientation towards international readership (Kristiansen et al. 2020). Kristiansen et al. (2020) selected one left-leaning and one right-leaning UK publication- The Guardian and The Telegraph. Carvalho also makes a case for using “quality newspapers” to explore climate change discourses, arguing that *“a debate on this complex issue is excessively simplified or excluded in other media”* (Carvalho 2007, p. 226). She asserts that her chosen broadsheets- The Guardian, The Times, and The Independent- play an important role in agenda-setting both for the public and other media sources; as well as being preferred by policymakers.

However, tabloid newspapers often have a wide readership among a range of socioeconomic groups (Mroz and Painter 2023), and are popular online. For example, MailOnline was used by 15% of respondents to a Reuters survey in 2020 (Newman et al. 2020), and in 2021 had significantly more Facebook followers than many broadsheet newspapers. We follow Mroz and Painter (2023) in arguing that these publications also play a role in meaning making and agenda-setting among the public, which, as for the quality newspapers, also includes ruminant livestock farmers and their communities.

Meanwhile, discourses may differ between national and industry-specific media. Morris (2018) found that regional newspapers were more likely than national newspapers to report meat reduction campaigns negatively, and paid more attention to impacts on the livestock farming sector. The author suggests this may be due to these publications having a more rural audience, and highlights the need to examine farming sector media as well as news media aimed at a general audience, *“as part of a wider research effort…that engages agricultural actors in deliberating the challenges of meat production and consumption”* (Morris 2018, p.448).

Building on previous research, this paper employs CDA across national and farming industry news media. The aims are to identify CDMs and examine comparatively how these sources contribute to the discursive landscape. This includes whether and how they apply discursive strategies like (de-)politicisation and (de-)legitimisation to assert norms and values.

## Methodology

### Selection of news media sources

Four national newspapers searchable on the LexisNexis platform were selected, guided by the approach taken in previous studies (Carvalho 2007; Khazaal and Almiron 2016; Kristiansen et al. 2020). To incorporate a range of perspectives, one left-leaning and one right-leaning quality newspaper (The Guardian and The Daily Telegraph), plus one left-leaning and one right-leaning tabloid (The Mirror and The Daily Mail) were selected. Newspaper selection for each category was based on having the highest multiplatform reach, while also considering numbers of Twitter and Facebook followers, given that these are the social media platforms most commonly used for news in the UK (Newman et al. 2020).

Farmers Weekly and Farmers Guardian were selected to represent UK farming media. Although there are many livestock sector magazines targeted at specific production systems (e.g., British Dairying, Sheep Farmer), general farming magazines tend to be more influential within the sector and have more followers on social media platforms. Furthermore, from a pragmatic perspective, the former tend to have less searchable archives and would require manual scanning of past editions to locate relevant articles.

### Search parameters

The selected date range for all nominated media sources was 1st January 2016 to 21st November 2021; a period of just under six years representing the “post Paris era” following the 2015 Paris Agreement (UNFCCC 2021), and including the UK presidency of the UNFCCC secretariat’s annual Conference of the Parties (COP26) in Glasgow in the final month (UK Government 2021a). This range also captures the start of the “Brexit era”, as the UK’s referendum on EU membership took place in 2016. The period from 2016 to 2021 was characterised by significant changes and uncertainty in the UK farming sector, particularly in relation to withdrawal from the EU’s farm subsidy scheme and design of a UK alternative scheme (Hubbard et al. 2018). Furthermore, the late 2010s saw publication of both the EAT/Lancet commission (Willett et al. 2019) and the IPCC’s Special Report on Climate Change and Land (Shukla et al. 2019). The former was a report on the planetary health impacts of the food system which prescribed a healthy and sustainable “global reference diet”. The latter was a report by the UN’s Intergovernmental Panel on Climate Change which focused primarily on how land interacts with climate systems and how unsustainable land use practices can drive climate change. As noted above, both publications provoked a lot of attention in traditional media and public discussion on social media (Sanford et al. 2021; Mroz and Painter 2023). Moreover, they were not captured in many previous analyses (Lee et al. 2014; Morris 2018; Kristiansen et al. 2020).

The following string was used to search the national newspapers using LexisNexis:

(“climate change” OR “global warming” OR carbon OR methane OR “greenhouse gases”) AND (livestock OR cattle OR cow OR sheep OR beef OR dairy OR ruminant).

Omission of the term “meat” was to minimise the number of articles not specifically discussing ruminants. Farming news media were searched using their individual websites with the phrase “climate change”, as search functions were not compatible with Boolean operators.

### Inclusion of newspaper articles and analysis

The headlines of returned articles were scanned, and those appearing relevant were read in full. Articles were included in the overall corpus if they met the following criteria:

- All article formats except readers’ letters.
- More than one sentence on the connection (or lack thereof) between ruminant livestock and climate change.
- UK focus, or other country with explicit reference to UK interests.

Articles were excluded if they focused entirely on the impact of climate change on animal agriculture with no mention of the opposite relationship.

A data journal was kept during this process to record emerging themes based on the initial reading, and to identify “Significant Happenings” and possible CDMs (Carvalho 2008). Once the corpus was complete, the number of articles published per month was plotted on a graph to identify periods of increased publication frequency, denoting increased salience. This information, triangulated with the notes kept during the inclusion process, identified 26 Significant Happenings. These events led to a higher than usual number of articles, as well as suspected discursive shifts. Articles pertaining to each Significant Happening were then collated and re-reviewed. An article was defined as belonging to a specific Significant Happening if it met the following criteria:

- More than a passing mention of the Significant Happening (> 1 mention).
- Within one month of the Significant Happening.

**Fig. 1:**
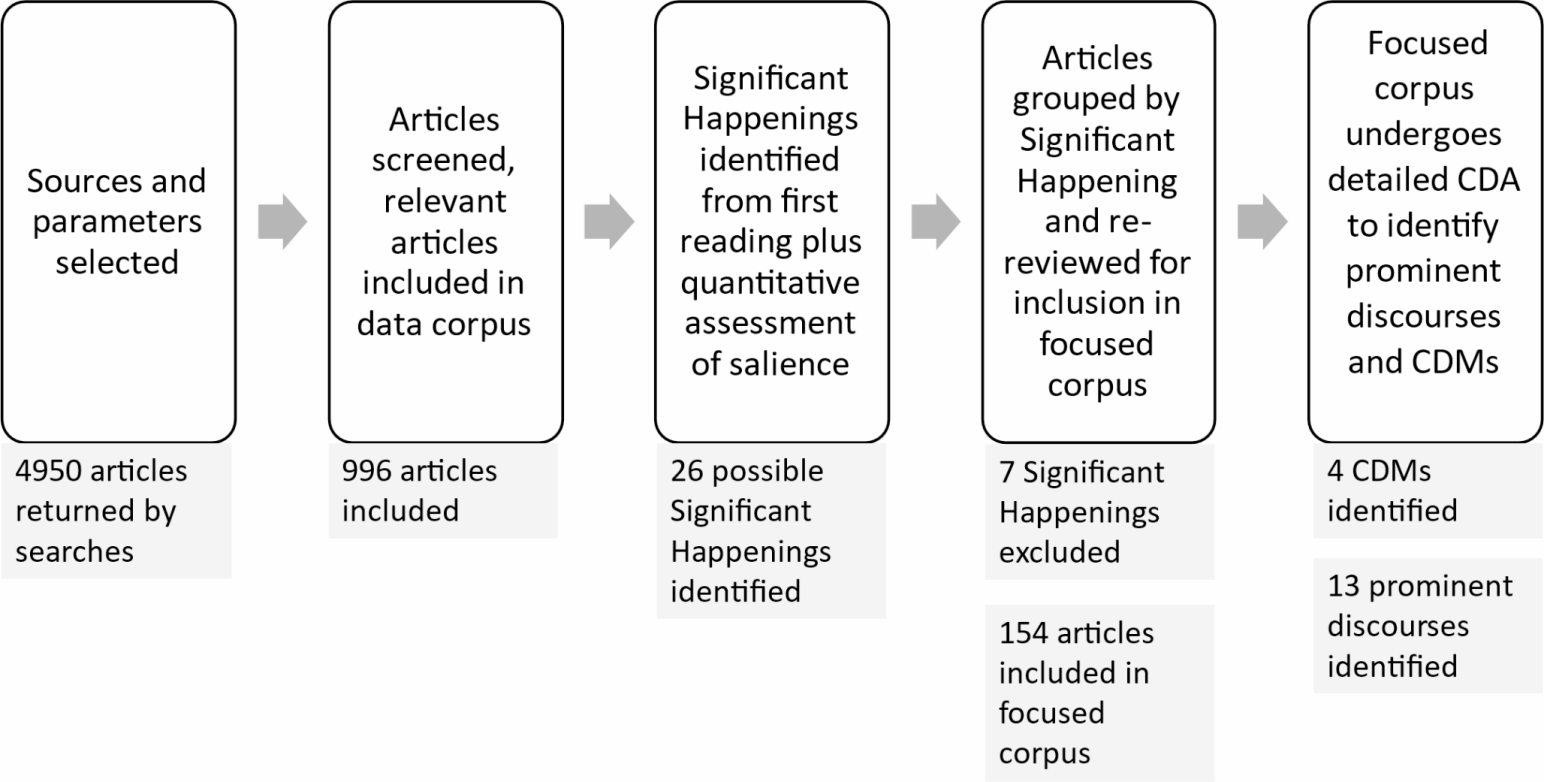
Search and analysis procedures to identify CDMs

Articles covering each of the 26 possible Significant Happenings were reviewed by the research team to assess whether new arguments emerged (Carvalho 2008)- at this stage seven were excluded, leaving 19 Significant Happenings for detailed analysis. The relevant articles were entered into NVivo and grouped in folders, then analysed according to Carvalho’s (2008) CDA framework (Table 2) to identify specific discourses and define CDMs over the 6-year period. Discourses were identified inductively via attention to language, grammar, and rhetoric, and the Discourses of Climate Delay (Lamb et al. 2020) were identified deductively. CDMs were defined when discourses emerged or changed across multiple articles. The process from search to CDMs is illustrated in Fig. 1.

The major discursive strategies we sought to identify were framing, (de-)politicisation and (de-)legitimisation. For Carvalho, framing refers to how an actor organises discourse “according to a certain point of view or perspective” (Carvalho 2008, p. 169). This differs from a more general framing analysis, in which framing is defined as repetitively presenting information in a certain way, meaning that certain ways of understanding a situation may be sidelined or silenced (Lockie 2006; Naylor et al. 2017). Politicisation is the attribution of a political nature to a situation, while legitimisation involves justifying an action or situation on the basis of normative or other reasons (Van Leeuwen and Wodak 1999). Comparative-synchronic analysis involved comparing articles from different sources covering the same incident. Historical-diachronic analysis involved accounting for contextual factors and examining the evolution of discourses over time (Carvalho 2008).

**Table 2:** Framework for analysis of media discourse, adapted from Carvalho (2008, p. 167)

Components of CDA	
Textual analysis	Contextual analysis
1. Layout and structural organisation 2. Objects 3. Actors 4. Language, grammar, and rhetoric 5. Discursive strategies 6. Ideological standpoints	1. Comparative-synchronic analysis 2. Historical diachronic analysis

## Results

In this section we present descriptive data on the news media corpus, followed by an overview of major patterns observed and CDMs identified. We then present detailed findings from each CDM, demonstrating emerging discourses and discursive strategies, and comparing national with farming news media.

### Overview of UK news media CDMs pertaining to ruminant livestock and climate change

The number of returned results and included articles for each source are shown in Table 3 below. The overall corpus totalled 996 articles, of which 660 were from national media sources and 336 from farming media sources.

**Table 3:** Number of search results, articles included in corpus, and articles analysed in depth

Search location	Results in date range	Included articles	Articles undergoing detailed CDA
National news media	3425	660	91
Farming sector news media	1525	336	63
Total	4950	996	154

**Fig. 2:**
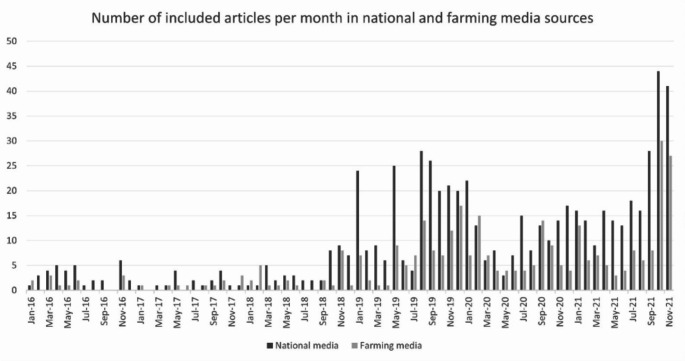
Number of included articles by month of publication

Over the almost six-year period, the topic of ruminant livestock and climate change grew in salience. Figure 2 shows the number of included articles by month of publication. It reveals a general upward trend, with a dramatic increase in January 2019 and the highest number of relevant articles being published in October and November 2021, around COP26. The trend is similar for national and farming sector media. For example, in January 2016 there were three relevant articles published across both media types. January 2017 and 2018 show similar numbers, then in January 2019 there were over 30 relevant articles published. In October 2021 this rose to over 70.

The following discourses were most prevalent in the analysed articles, appearing in over 30 articles: “Meat is harmful to planetary health” (59 articles), “Us vs. them” (50 articles), “UK livestock farming is efficient and sustainable” (38 articles), “Livestock farming is part of the solution to climate change” (37 articles), and “Radical restraint is necessary” (36 articles). See Supplementary Table 1 for further details. Of these discourses, the first and last were more common in the national media, while the rest were more common in the farming media. Among the Discourses of Climate Delay, the most prevalent were: “Whataboutism” (30 articles), “Individualism” (30 articles), and “Technological optimism” (17 articles). A list of all discourses appearing in five or more articles can be found in Supplementary Table 1.

**Fig. 3:**
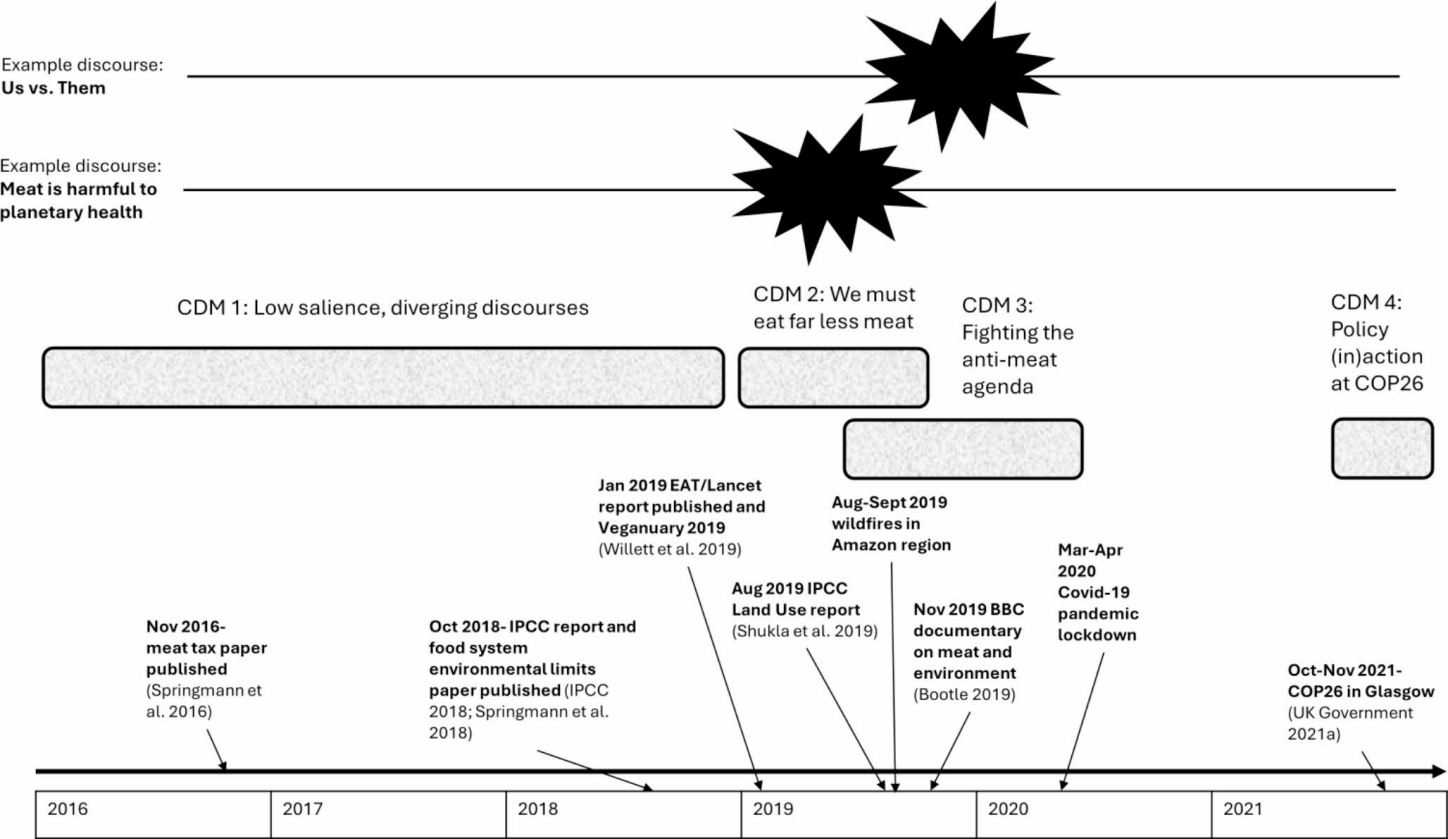
Timeline of CDMs, showing selected significant happenings and examples of discourses becoming prominent

Analysis of the media corpus identified four Critical Discourse Moments (CDMs): (1) Low salience, diverging discourses, (2) We must eat far less meat, (3) Fighting the anti-meat agenda, and (4) Policy (in)action at COP26. An overview of the CDMs is shown in Table 4, indicating their timescale, context, and site (national and/or farming sector news media). The CDMs are somewhat leaky, in that their prominent discourses often emerged briefly earlier in the corpus, then were rearticulated as they became more salient. This is illustrated in Fig. 3, which shows two example discourses that were identified throughout the corpus but became prominent during specific CDMs. For each CDM, we identified prominent discourses through assessing their salience (number of articles containing the discourse), plus their novelty (whether this was the first time the discourse had emerged) or rearticulation (when the discourse had emerged earlier but shifted in response to a Significant Happening).

It is also important to note that CDMs overlap temporally. CDM 2 (We must eat far less meat) begins in early 2019, predominantly in the national media, while CDM 3 (Fighting the anti-meat agenda), which is more prominent in the farming media, begins seven months later (Fig. 3). Discourses pertaining to both had already emerged previously in a more scattered way, but the two CDMs became distinct in response to Significant Happenings, as described below.

**Table 4:** CDMs and their characteristics

Name	1. Low salience, diverging discourses	2. We must eat far less meat	3. Fighting the anti-meat agenda	4. Policy (in)action at COP26
Timescale	Jan 2016– Dec 2018	Jan 2019– Sept 2019	Aug 2019– Apr 2020	Oct - Nov 2021
Summary	Low salience punctuated by publication of scientific papers and reports.	Initially associated with EAT/Lancet report and CCC Net Zero report, reiterated around e.g. university “beef ban” and 2019 Amazon fires.	Commences with publication of IPCC Special Report on Climate Change and Land, and runs parallel to CDM 2, reiterated around e.g. BBC documentary “Meat: A threat to our planet?” and Covid-19 lockdown.	Increased issue salience around publication of UK’s Net Zero strategy and COP26 in Glasgow.
Site	Divergent discourses in national and farming sector media.	Predominantly in national media.	Predominantly in farming media.	Similar salience in national and farming media, discourses remain largely polarised.

### CDM 1: Low salience, diverging discourses

CDM 1, summarised in Table 5, describes approximately the first three years of the study period. It is characterised by low overall salience, punctuated by occasional publications that received some media attention. Discourses emerged that were then reiterated or became more salient later in the corpus. For example, “Meat is harmful to planetary health” is the discourse repeated in the highest number of analysed articles (59 articles) throughout the entire corpus. In this first CDM, it mainly involves national media reporting of the ways meat production may impact climate and other aspects of planetary health:

The research, published in the journal Nature Climate Change, evaluated the tax required for each food type to compensate for the climate damage its production causes. Beef has a heavy footprint, due to the deforestation and methane emissions associated with cattle and the grains they are fed, and needed a 40% tax on average across the world. The Guardian, 7th November 2016

In this quotation, the article’s author legitimises the research in question by mentioning its publication in Nature, a respected academic journal. They also report on global averages, and imply that cattle are universally grain-fed, obscuring the UK prevalence of grass-fed ruminant livestock and the role of pigs and poultry in consuming grain. The verb “needed” in reference to the “40% tax” implies acceptance of taxation as a necessary part of governance.

**Table 5:** Significant happenings and prominent discourses in CDM 1

Significant happenings	Nov 2016- meat tax paper published (Springmann et al. 2016)	May 2017- eating insects paper published (Alexander et al. 2017)	May 2018- food system environmental impacts paper published (Poore and Nemecek 2018)	Oct 2018- IPCC report and food system environmental limits paper published (IPCC 2018; Springmann et al. 2018)	Nov 2018- CCC land use report published (Climate Change Committee 2018)
Prominent discourses	“Meat is harmful to planetary health”, “Us vs. them”.				

In CDM 1 we also saw diverging discourses in the national and farming sector media. For example, this quotation and one below are both reporting on the same scientific publication:

Government and campaign groups have had little appetite for tackling the overconsumption of meat, but Alexander said awareness of the issue is growing rapidly, though action is slow. “We are moving slowly in the right direction,” he said. The Guardian, 5th May 2017

Despite emerging early in the corpus, the discourse “Politicians are not willing to act on meat reduction” remained quiescent for a few years, re-emerging only in 2021 when the UK published its Net Zero strategy. In the above quotation, “the overconsumption of meat” is treated as an accepted fact, while the issue is politicised through the mention of “government”. However, this CDM also features a strong “Individualism” discourse (a Discourse of Climate Delay), with a focus on individual dietary changes rather than systemic approaches.

Several new discourses also emerged in the farming media:

She said: Many things could help reduce climate change attributed to emissions, such as reducing dependence on the combustion engine or cutting use of plastics, but it is a sad fact that often it is meat eating that is in the firing line. Farmers Guardian, 10th May 2017

The use of metaphors such as “hit back”, “attack”, and “firing line” are prevalent throughout the corpus, especially in the farming media, creating an image of the ruminant livestock sector as a victim defending itself from violence. This contributes to an “Us vs. them” discourse, in which we see increasing construction of two distinct “sides” in a metaphorical battle. The quotation above is also the first example of “Whataboutism” in the corpus, with the speaker redirecting attention to fossil fuels via “the combustion engine” and plastics. This discourse remained common, especially in the farming media, as we will demonstrate below.

### CDM 2: We must eat far less meat

CDM 2, summarised in Table 6, coalesced from January to September 2019. It began at the time of the publication of the EAT/Lancet commission report in January 2019, and was subsequently reiterated in various ways, most notably with the fires in the Amazon region of Brazil in summer 2019. Publication of the EAT/Lancet commission and its “global reference diet” resulted in a large increase in salience of the “Radical restraint is necessary” discourse, which had emerged briefly in the previous CDM:

Globally, the diet requires red meat and sugar consumption to be cut by half, while vegetables, fruit, pulses and nuts must double. But in specific places the changes are stark. North Americans need to eat 84% less red meat but six times more beans and lentils. For Europeans, eating 77% less red meat and 15 times more nuts and seeds meets the guidelines. The Guardian, 16th January 2019

The above quote, from The Guardian, shows that the “we” in this CDM’s title, “We must eat far less meat”, refers to people in high-income “Western” countries such as the UK. The journalist reports the diet factually in this quotation, with limited emotion, though the word “stark” suggests these changes are viewed as radical. In contrast, national tabloids were more likely to explicitly de-legitimise the proposal:

Full English to fool’s English? In place of a full English, the report suggests brown rice, peas, half a baked potato, a quarter of a rasher, spinach and red pepper. Really? Daily Mirror, 17th January 2019

In this quotation we see the radical restraint discourse being framed in terms of an attack on traditional values, in which meat is linked to “Englishness”. The rhetorical question serves to further delegitimise the reference diet, which was published as a daily weight for each food type. This was taken literally by some journalists, especially those who were sceptical, with reporting of daily allowances of e.g. beef making the diet sound more restrictive than extrapolating to describe a typical week or month.

**Table 6:** Significant happenings and prominent discourses in CDM 2

Significant happenings	Jan 2019 EAT/Lancet report published and Veganuary 2019 (Willett et al. 2019)	May 2019 CCC Net Zero report (Climate Change Committee 2019)	Aug 2019 IPCC Land Use report (Shukla et al. 2019)	Aug 2019 Goldsmiths removes beef from campus food outlets (Wilson 2019)	Aug-Sept 2019 wildfires in Amazon region
Prominent discourses	“Meat is harmful to planetary health”, “Radical restraint is necessary”, “Imported foods are worse for the environment”. “Individualism”.				

This Significant Happening also saw the emergence of a new discourse: “Climate campaigners are hypocrites”:

…The head of lifestyle economics at the Institute of Economic Affairs– said: The hypocrisy of this is breathtaking. This is a campaign telling ordinary people they should be eating less than half a rasher of bacon per day for the sake of the environment, while the patron is flying people around the world in private jets creating one enormous carbon footprint. Daily Mirror, 18th January 2019

In this instance, a left-leaning tabloid quoted a spokesperson from a free market think tank to comment on the reference diet. The words “ordinary people” imply one of the discourses of climate delay: an “Appeal to social justice”. In this situation the discourse is deployed cynically, to advocate the organisation’s distaste for any kind of top-down governance. We also see a reprise of “Whataboutism”, with the focus on aviation emissions of the EAT/Lancet report’s funder.

Meanwhile, the discourse “Proposed government action on methane emissions is suspicious and misguided” appeared in nine articles, first emerging in response to the CCC’s May 2019 Net Zero report:

Last night the Government backed the radical plans drawn up by the influential Committee on Climate Change, saying it would legislate to make Britain the first major world economy to reduce its carbon footprint to zero by 2050. Daily Mail, 2nd May 2019

Concerns were raised later in this article that the plans would be “economic suicide”, transitioning to a “Whataboutism” discourse in which the idea of reducing UK emissions ahead of other countries is foolish, and quoting a stakeholder from a free market think tank. The report was also described as “stark” and “dramatic”. We therefore interpret the use of words like “radical” to imply that the Net Zero report was unusually severe, and that the journalist may be sceptical of the CCC’s “influential” relationship with the government. This interpretation is also informed by the article being published in a right-leaning newspaper, and thus likely to be aligned with their ideological standpoint.

A Significant Happening that only featured articles from the national media was the August 2019 “beef ban” at Goldsmiths, University of London. This was notable because several articles framed the issue by organising discourse to suggest a “culture war” perspective. One article led with the headline “Now snowflakes ban beef burgers”, using the pejorative term for (often young) people who are believed to be overly sensitive. Another article ended with a timeline of “University crackdowns”, such as “A pro-life student group was barred from three different freshers’ fairs”. These articles implicitly associated climate concerns around beef with other progressive values within the so-called culture war, and therefore a potential threat to conservative values.

Towards the end of CDM 2, media responses to the Amazon fires in summer 2019 further cemented the “we” in “We must eat far less meat”. The discourse “Imported foods are worse for the environment” became salient, repeatedly making explicit the connection between deforestation in the Amazon and UK consumption habits:

It’s all very well for European governments to condemn Bolsonaro, but western demand for Brazilian beef is contributing to deforestation. The EU imported more than £490m worth of beef from Brazil last year. Consumers in Britain were indirectly responsible for the destruction of the equivalent of 500 football pitches of rainforest in Brazil last year… The Guardian, 25th August 2019

This responsibilisation of UK consumers errs on “Individualism”, though later in the article the journalist highlights the EU-Mercosur trade deal as a lever to negotiate enhanced rainforest conservation, thus politicising the issue. The discourse “Imported foods are worse for the environment” was used to critique plant-based foods as well as ruminant products from overseas.

Across the corpus, there was a broadly equal split between the “Individualism” discourse, and the discourse “Systemic solutions are needed to address climate impacts of ruminants”. The former of these was somewhat more prevalent during CDM 2, especially in the earlier months due to its association with the “Radical restraint is necessary” discourse illustrated in some of the quotations above. However, one discourse notable by its scarcity was “Big Food is powerful”. This only appeared in one article (in The Guardian), which mentioned “powerful vested interests and misplaced economic incentives” as well as “dark money”. Although many articles referenced the need for government action and redirection of subsidies to improve the food system, few highlighted the power corporations hold to maintain the status quo, or imagined solutions beyond market-based incentives and subsidies.

### CDM 3: Fighting the anti-meat agenda.

CDM 3 is shown in Table 7 as spanning October 2019 to April 2020, though in fact it began earlier and ran parallel to the previous CDM (shown in Fig. 3), primarily in the farming sector media. Many of its key discourses were present early in the corpus, but their salience only reached a critical mass with publication of the August 2019 IPCC Land Use report, shown in Table 6 under CDM 2. At this point, the farming media generated a significant shift in the “Us vs. them” discourse:

Rather than letting the likes of NFU president Minette Batters spend their time tackling the anti-farming brigade on Twitter, it is time for the unions, representative bodies and levy boards to come together to form an action plan which seeks to properly communicate the proactive contributions of British farming, both at an environmental and societal level. Farmers Guardian, 16th August 2019

In this quotation, the journalist moves from discussing an “anti-meat agenda” (a popular phrase in earlier articles) to an “anti-farming agenda”. The object to be defended therefore shifted from the product (meat) to the people (farmers).

The above quotation was in response to national media reporting of the IPCC report, which led with headlines on meat reduction rather than the report’s more nuanced recommendations. This led to strong reiteration of the discourse “The media is biased and spreading disinformation” in the farming media, which persisted through the rest of this CDM. It also prompted repetition of the discourse “Livestock farming is part of the solution to climate change”, which was present throughout the corpus:

NFU president Minette Batters said: Having gone through the report in detail, it is clear that the IPCC recognises the important role animal products play in a balanced diet, and when produced sustainably in low greenhouse gas-emission systems, it is actually part of the solution to climate change. Farmers Weekly, 9th August 2019

The NFU president represented a key actor who was regularly quoted on the topic of methane emissions from ruminants in the farming media as well as (less frequently) in national media.

**Table 7:** Significant happenings and prominent discourses in CDM 3

Significant happening	Oct 2019 Animal Rebellion action in London meat market	Nov 2019 BBC documentary on meat and environment (Bootle 2019)	Feb 2020 Backlash to Veganuary 2020	Mar-Apr 2020 Covid-19 pandemic lockdown
Prominent discourses	“Us vs. them”, “UK livestock farming is efficient and sustainable”, “Livestock farming is part of the solution to climate change”, “The media is biased and spreading disinformation”, “Whataboutism”, “Farmers’ mental health is negatively impacted by these debates”.			

An October 2019 action by activist group Animal Rebellion (a blockade of a meat market in London) showed strong salience of “Climate campaigners are hypocrites”, as well as “Whataboutism”:

Climate change campaigners forced meat traders out of London’s most iconic meat market and replaced them with stands of imported fruit and vegetables. Industry members on the scene photographed pears from Belgium and salad leaves from France, suggesting they had ‘less than desirable’ air miles. Farmers Guardian, 9th October 2019

The journalist never mentions the name of the group (an explicitly vegan wing of Extinction Rebellion), choosing instead to label them generic “climate change campaigners”. This shows the discursive construction of a poorly defined opposition that may include anyone involved in climate campaigns. The fact they “forced” entry to the “iconic meat market” implies a dangerous incursion on valued cultural spaces, while the focus on air miles is identified as “Whataboutism”, with a “Climate campaigners are hypocrites” flavour.

A BBC documentary on the environmental impacts of meat in November 2019 resulted in a strong backlash from the farming media, plus a couple of articles in the national media echoing their stance. This Significant Happening featured a strong reprisal of the discourse “UK livestock farming is efficient and sustainable”, one of the most prevalent in the corpus:

It has been frustrating to see the continued media portrayal of red meat production as the same throughout the world– especially when the truth is that British red meat is some of the most sustainable in the world, produced by farmers who care– with a greenhouse gas footprint 2.5 times lower than the global average, he added. Farmers Guardian, 25th November 2019

The “2.5 times lower” statistic was often repeated, illustrating a focus on the relative emissions of different products (emissions per unit) rather than absolute emissions, which account for the level of consumption. Though this discourse was often repeated, and even used to disparage farming in other countries (especially those striking trade deals with the UK and thus risking ruminant livestock sector profits), one article extended solidarity to Brazilian farmers following the BBC documentary, quoting two of them describing their environmentally friendly practices. The above quotation works to legitimise British farmers by saying that they “care”, implicitly about the environment as well as their animals.

The next Significant Happening was a spate of articles in February 2020 in response to that year’s ‘Veganuary’ campaign (a public engagement initiative that encourages people to try veganism for the month of January). Here, discourses in the “Fighting the anti-meat agenda” CDM became more organised and cohesive, in terms of speaking openly about the emotional impacts of the “anti-farming agenda”, and agenda-setting on three fronts: reducing emissions, refuting accusations, and educating the public. In terms of emotional impacts, the discourse “Farmers’ mental health is negatively impacted by these debates” emerged here:

The issue of meat consumption has been particular [sic] sensitive for farmers. This week the National Farmers’ Union (NFU) said that the “anti-meat agenda” had become an added strain on farmers’ mental health and that the demonising of livestock farmers was having “real-life consequences”. The Guardian, 27th February 2020

This discourse was identified in three national media articles, as well as being salient in the farming media, with a connection to high suicide rates often being drawn. In the above quotation the issue is broadly depoliticised, with a focus on “anti-meat” rhetoric rather than rural mental health services or support for transitioning away from EU farming subsidies. Furthermore, “anti-meat agenda” is in scare quotes, implying that the article’s author was sceptical of the concept, wishing to attribute it to the NFU rather than claiming it as fact (as was the norm in the farming media). While articles responding to previous Significant Happenings often described farmers as “angry” or indeed “furious”, this was the first time in the corpus that articles discussed more feminised emotions such as sadness and vulnerability. The people quoted in these articles were mainly women, with any male farming sector actors tending to reproduce more longstanding discourses about being “under attack”. Thus, at this time there still appeared to be a reticence among men within the ruminant livestock sector to speak openly about the mental health impacts of this public debate, at least with journalists.

The final Significant Happening in CDM 3 is the onset of the Covid-19 pandemic in March-April 2020. This event was related to ruminant livestock and climate very differently by the national and farming media. In the national media, two articles in The Guardian argued that meat consumption increases the risk of pandemics, due to agricultural expansion bringing humans into greater contact with wild animals. This is a reiteration of the discourse “Meat is harmful to planetary health”, and thus a calcification of CDM 2.

Meanwhile, in the farming media, the reduction in air pollution resulting from Covid-19 lockdowns generated a “Whataboutism” discourse:

The farm sits under a flight path to Stansted Airport and, since the lockdown began, with nearly all flights grounded, [farmer’s name] has noticed the difference in air quality, which makes her question whether farmers are shouldering an unfair amount of blame for the climate crisis. Farmers Weekly, 1st May 2020

This was reproduced across several articles in the farming media, with industry stakeholders arguing that reductions in air pollution proved that ruminant livestock were not contributing to climate change. This conflated data on nitrogen dioxide and particulate matter with methane. The data in question, from the National Centre for Atmospheric Science, was corroborated, and thus legitimised, by sensory experiences of farmers working near airports and busy roads. In the above quotation we can see reiteration of “Us vs. them”, in terms of the “unfair…blame” placed on farmers. We also see that the blame is experienced as being placed on the people (farmers) rather than on the product or the animals.

### CDM 4: Policy (in)action at COP26

The fourth and final CDM, summarised in Table 8, occurred at the end of the study period with a high volume of relevant articles, especially around the United Nations’ 26th Conference of the Parties (COP26). It signifies a “political crunch time”, in which we see a mix of political action and inaction in response to opportunities to legislate around ruminant livestock and climate.

**Table 8:** Summary of significant happenings and prominent discourses in CDM 4

Significant happenings	Oct 2021- UK Net Zero Strategy published (UK Government 2021b)	Oct-Nov 2021- COP26 in Glasgow (UK Government 2021a)
Prominent discourses	“Methane reduction is an opportunity to mitigate climate change”, “Politicians are not willing act on meat reduction”, “Systemic solutions are needed to address climate impacts of ruminants”.	

The October 2021 publication of the UK’s Net Zero Strategy ahead of COP 26 provoked a slew of relevant articles, mainly in the mainstream media. Right-leaning newspapers focused on potential costs to the public, while left-leaning newspapers argued the strategy was inadequate to meet climate goals. In particular, the lack of legislation on food and aviation were critiqued. Meanwhile, the UK Government’s Behavioural Insights Team, at that time part of the civil service, published a report online that was swiftly deleted again. The report contained proposals for regulatory measures such as taxation on meat and frequent flyers, leading to a resurgence of the discourse “Proposed government action on methane emissions is suspicious and misguided” in news media. Meanwhile, “Radical restraint is necessary” was reiterated, with left- and right-leaning newspapers suggesting that climate action going forward would require behaviour change among the general public. The discourse “Politicians are not willing act on meat reduction”, quiescent since CDM 1, made a strong comeback:

The Government said: “This was an academic research paper, not government policy. We have no plans to dictate consumer behaviour in this way. Our net zero strategy published yesterday contained no such plans. The Daily Telegraph, 21st October 2021

The word “dictate” implies an ideological standpoint in which government intervention is undesirable and an impingement of personal freedom. Repetition of “no…plans” reinforces this vehement disavowal of the report’s suggestions. This incident seems to support stakeholders’ arguments back in CDM1, suggesting that in 2021 there was also a lack of political will for regulation to promote meat reduction.

The second Significant Happening in this CDM was COP26, which took place in the UK. One of the outcomes was a Global Methane Pledge for voluntary reductions in methane emissions, led by US President Joe Biden and signed by over 100 countries. This resulted in a new discourse, “Methane reduction is an opportunity to mitigate climate change”:

The joint initiative, launched by US and European leaders, will tackle the potent greenhouse gas which is crucial to keeping warming limited to 1.5 C. It is also one of the fastest ways to reduce global warming. Daily Mail, 3rd November 2021

This article, in a right-leaning newspaper, uses crisis rhetoric such as “crucial” to suggest the pledge is urgent and will be effective. It also highlights the role of “US and European” leaders, and several articles highlighted that China, India, and Russia had not signed. The pledge was therefore framed as being delivered by “Western” (and therefore capitalist) actors despite non-cooperation from ideological antagonists. This contrasts with ideological framings in response to university beef bans, which associated meat reduction with leftist concerns, and in one case specifically with communism. As methane arises from various sources including fossil fuels, it’s possible that the Global Methane Pledge was more ideologically acceptable than focusing only on emissions from ruminant livestock.

Meanwhile, the implications of the methane pledge for ruminant livestock were discussed, with some concerns raised and some reassurance that the pledge would not cause excessive impacts:

NFU president…told the Farmers Weekly podcast: The global methane pledge will cause concern in its name, but it is primarily focused on fracking, oil and coal sites. It’s a chance for us to talk up the solutions on how we do lower methane [emissions in livestock]. 3rd November 2021

Here, the discourse “Livestock farming is part of the solution to climate change” is reiterated, as well as “The livestock sector needs to speak out more”. Meanwhile, another article focused on methane-reducing feed additives that were not yet licenced for use in the UK, an example of “Technological optimism” (a Discourse of Climate Delay).

The discourse “Systemic solutions are needed to address climate impacts of ruminants” was more salient relative to the “Individualism” discourse in CDM 4:

…They said farmers should be supported in partnership with government and industry to produce more with less environmental impact. It is vital that agriculture, land use and biodiversity policies are practical and properly funded, with a portfolio of measures across many different farm types. Farmers Weekly, 4th November 2021

Systemic solutions mentioned in left-leaning national media were usually in the form of regulation (such as taxes) or sometimes redirection of agricultural subsidies, while the farming media were more likely to highlight reform of agricultural subsidies and advocate farmers being paid for environmental activity.

Overall, despite political action at COP26 leading to a flurry of relevant articles, the methane pledge was not specifically focused on ruminants. Neither side of the polarised debate got exactly what they wanted: cattle and the farming sector were still highlighted as emitters of methane, but not specifically targeted by UK legislation. Rather, ruminants were associated with technical solutions that aimed to maintain current production and consumption levels.

## Discussion

This paper has examined how UK news media portrayed ruminant livestock’s impact on climate change between 2016 and 2021. Our results demonstrate significant differences between mainstream and farming sector news media, with discourses in the former provoking a backlash in the latter. By taking a qualitative approach using CDA, this study builds upon previous research (e.g. Morris 2018; Kristiansen et al. 2020; Rust et al. 2021) and adds further depth to our understanding of how news media can represent the relationship between ruminant livestock and climate change. In particular, by using a temporal approach to identify four critical discourse moments (CDMs) between 2016 and 2021, this research makes a novel contribution to agri-food knowledge by illustrating when and how in this time period polarisation between mainstream and farming news media increased. These findings and the methodology are likely to be relevant in other industrialised countries with similar news media landscapes and ongoing public debates about ruminant livestock and climate change. The discussion turns firstly to summarise the key findings and their alignment with previous research on media discourses and polarisation, including implications for policymakers and practitioners, and then to offer potential directions for future research.

### Ruminant livestock and climate change issue salience

Our data revealed a dramatic increase in issue salience for the climate change and ruminant livestock nexus, commencing around halfway through the study period, which aligns with research emerging from the UK and other high income countries (Mroz and Painter 2023; Saville et al. 2024). The corpus ended at the time of a headline-grabbing global pledge to limit methane emissions, and the continued association of cattle with methane, but little tangible change at UK scale. Neither proponents of CDM 2 nor CDM 3 got exactly the political action they hoped for. A recent systematic review demonstrated that the 2019 EAT-Lancet Commission has been highly influential in academia since its publication (Tulloch et al. 2023), and our research shows that it was also influential in UK news media, contributing to a significant discursive shift. This contributed to provoking a strong defence from the farming press, echoing their response to the animal rights movement in the late 20th century (Reisner 1992).

It is important to note that the identified CDMs are leaky, with discourses often emerging early in the corpus, and not becoming salient until a later Significant Happening. Their leakage extends also between national and farming sector media, with the former sometimes contributing to CDM 3- “Fighting the anti-meat agenda”, and the latter sometimes reproducing discourses such as “Meat is harmful to planetary health”. Another apparent, yet leaky, distinction was between left-leaning and right-leaning national media. The former were more likely to focus on discourses such as “Radical restraint is necessary”, while the latter were more likely to reproduce “culture war” framings. This framing aligns with a recent analysis of media responses to England’s National Food Strategy (Tak et al. 2024). In terms of specific newspapers, The Guardian had the highest number of included articles overall, reflecting its focus on climate journalism (Kristiansen et al. 2020).

### Role of media analysis in understanding societal debates

Conti et al’s (2021) systematic review of barriers to change in agri-food systems identified that attitudes and cultures can cause aversion to change. The media plays a key role in creating, reproducing, and contesting societal meanings (Burgess 1990; Corbett and Durfee 2004; Carvalho 2008), and can promote attitudes and cultures that reinforce the status quo (Neff et al. 2009; Friedlander et al. 2014; Almiron and Zoppeddu 2015). Traditional news media outlets still play a significant role in agenda setting (Carvalho 2007; Friedlander et al. 2014; Happer and Wellesley 2019), despite increased prominence of social media for sharing news. Furthermore, media discourses can have real world consequences. For example, researchers have highlighted how news media and policymakers in Ghana reinforced lock-in to prior decisions on agri-food development policy, as factories became imbued with symbolic and political value despite economic failure (Frimpong Boamah and Sumberg 2019).

While media analysis can be highly valuable for understanding social norms and narratives, it is difficult to ascertain whether the media is the source of new ideas, or simply reflecting those originating from readers. The reality is likely a bidirectional influence, with news media and public discourse contributing to issue framing (Zhou and Moy 2007). For example, a study of US news media coverage of an oil spill found evidence of “reverse agenda setting”, where the news media responded to online search trends, indicating that influence is a “two way street” (Ragas et al. 2014). In the UK, a qualitative analysis of farming sector media and interviews with farmers demonstrated that both presented sustainable agricultural practices within agronomic or economic frames more commonly than within environmental frames, indicating a shared worldview (Rust et al. 2021). Meanwhile, people are more likely to read articles that support their dietary habits, reducing the potential for media discourses to change attitudes (Lueders et al. 2022). It is therefore reasonable to suggest that the discourses represented in this paper may be broadly aligned with the perspectives of readers, and therefore may provide insights into areas of agreement and disagreement between social groups.

### Alignment with prior research on de-meatification, response scepticism, political economy of food system transformation, and polarisation

Despite previous research findings that show national news media’s reinforcement of animal agriculture, our findings align with research that identifies a process of “de-meatification” taking place in UK national media (Morris 2018; Mroz and Painter 2023), at least in relation to climate change. Although we did not quantitatively compare individual national newspapers, patterns align with evidence that left-leaning media publish more climate change coverage than right-leaning media (Carvalho 2007; Kristiansen et al. 2020). Members of the public who exclusively read right-leaning newspapers are therefore less likely to see coverage of these issues.

Meanwhile, our findings align with existing research to suggest that climate change denial is becoming less prevalent in news media, with a shift towards “response scepticism”: seeking to delay policy response (Painter et al. 2023). In some countries, this is resourced by a network of conservative advocacy organisations, foundations, and think tanks (Painter et al. 2023). All forms of climate scepticism tend to be more prevalent in right-leaning media (Carvalho 2007; Painter et al. 2023), a trend which was reflected in our data. We also found a higher prevalence of response scepticism in farming sector newspapers. Considering Lamb et al’s (2020) Discourses of Climate Delay, the most prominent were “Whataboutism”, “Individualism”, and “Technological optimism”. The analysis presented in this paper confirms that ruminants’ relationship to climate change remains prominently contested, likely more so than fossil fuels. This resonates with Sanford et al’s (2021) analysis of responses to the 2019 IPCC report on Land Use posted on Twitter. The authors found high levels of contention on the topics of dietary choices and the credibility of the IPCC, and concluded that the nexus of animal agriculture and GHGs had “strongly entered the climate change arena” (Sanford et al. 2021, p.59).

The systematic review by Conti et al. (2021) analysing barriers to change in agri-food systems identified that political economy factors skew the direction of change. Using CDA helped to explore this dimension, and to recognise that while national media may have often been supportive of changing the status quo away from high meat consumption, and both media types highlighted systemic solutions to methane emissions from ruminants, they tended to do so within a neoliberal framework of market incentives and subsidies. Powerful food system actors such as retailers were often overlooked, and proposed solutions were often reduced to individualistic approaches, such as eating less meat or buying certain kinds of meat, as identified in previous research (Kristiansen et al. 2020). Framing ruminant meat reduction as an impingement of personal freedom and traditional values was also prevalent in some newspapers, consistent with research across high-income countries (Sievert et al. 2022). Furthermore, the voices of consumers, particularly those living in poverty, were rarely included (Tak et al. 2024). Using CDA has therefore helped us to identify that despite a likely process of “de-meatification” in UK mainstream media, there is little appetite to regularly highlight powerful agri-food interests, nor to imagine or engage with more transformative changes that go beyond neoliberal governance and consumer responsibility.

Using CDMs to adopt a temporal approach to this analysis has also increased our understanding of the polarised nature of debates around climate and ruminant livestock in news media. We documented the emergence of a salient meat reduction message in national media, and a subsequent backlash in farming sector media. This demonstrated opinion polarisation between the two media types. We also identified the strong presence of an “Us vs them” discourse, and the use of discursive strategies to de-legitimise opposing speakers. These characteristics may have contributed to intergroup polarisation; the variety of polarisation most likely to contribute to social conflict (Judge et al. 2023). Furthermore, framing debates on emissions from ruminant livestock as part of a broader “culture war” likely contributed to polarisation (Tak et al. 2024). Policymakers are likely to be especially risk-averse when they perceive polarisation to be high (Howlett 2014), and if media discourses increased actual or perceived polarisation among readers, they also may have worked to inhibit collective action to progress food system transformation (Judge et al. 2023; Tak et al. 2024).

Judge et al. (2023) suggest several strategies for reducing actual and perceived polarisation. Polarisation can arise when the distributive aspects of environmental policy are overlooked- for example, when a group feels they will be disproportionately impacted by new policies. There is evidence that a shift to sustainable diets would disproportionally impact regions specialised in livestock production (Lehtonen et al. 2022; Rieger et al. 2023). For policymakers, taking a “just transition” approach to decarbonisation may help reduce polarisation by avoiding unfair impacts (Reay 2020; Judge et al. 2023). Other strategies for reducing polarisation include reframing and emphasising co-benefits of environmental policies to align with different value systems, involving citizens in deliberative processes (Willis 2020), and correcting individuals’ misperceptions about other groups in society (Lees and Cikara 2021). Policymakers and practitioners might find these insights useful for predicting press reactions to future high-profile publications, and creating pre-emptive strategies (Lewandowsky and van der Linden 2021) to mitigate polarisation and advance collaboration between diverse groups. Journalists also have a role to play in conveying the complexity and nuance of food system challenges, including a range of voices, and avoiding perpetuating inter-group polarisation. The use of “clickbait” headlines may exacerbate fears and biases, reducing the potential for nuanced and respectful debate (Rousseau 2021).

### Future research directions

Future research could investigate discourses emerging from “alternative” agricultural movements and media, thus going beyond mainstream farming media. Media data could also be triangulated with interviews with journalists and news editors, to identify mechanisms that perpetuate individualist discourses around climate change, and approaches that can better support journalists in their role as translators of climate change research. Overall, research that explores how to ameliorate algorithm-mediated polarisation on climate change is likely to be beneficial, including areas such as changes to link recommendation algorithms (Santos et al. 2021), deliberative processes (Willis et al. 2022), and strategies to reduce perceived polarisation (Lees and Cikara 2021). These approaches might also help to mitigate “cultural loneliness” in the farming sector and support collaboration between farmers and non-farmers (Wheeler et al. 2022).

## Conclusion

This paper has applied Carvalho’s (2008) CDM approach to critically examine UK media discourses in the ruminant livestock and climate change debate between 2016 and 2021. This represents a time in which conversations about methane emissions from ruminant livestock became more mainstream. While analysing media does not allow researchers to assume the impact on readers, it does enhance our knowledge of how the media seeks to shape the agenda and legitimise certain courses of action. In this respect it represents a transferable methodology to other countries and contexts. Using CDMs enabled deeper insights than a snapshot content analysis might have provided, revealing the evolution of significant differences between national and farming sector media, in the context of major societal events both internationally and in the UK, including Brexit and the Covid-19 pandemic. Overall, we identified polarisation between mainstream and farming sector media which may have impeded capacity for collective action, and found that media discourses in this area often contributed a limited focus on personal choices around meat consumption and pricing mechanisms, falling short of imagining transformative solutions to food system power imbalances.

## Supplementary Information

Below is the link to the electronic supplementary material.

Supplementary material 1 (DOCX 21.5 kb)

## Abbreviations

GHGs: Greenhouse gases
CDA: Critical discourse analysis
CDMs: Critical discourse moments
NFU: National Farmers’ Union
