# Supplementary material for: Ruminant livestock and climate change: critical discourse moments in mainstream and farming sector news media
Source: Agric Human Values. 2024 Nov 11;42(2):945–64. doi: 10.1007/s10460-024-10651-7 (PMC12098180; doi:10.1007/s10460-024-10651-7)
Supplement: Supplementary file 1 — Supplementary material 1 (DOCX 21.5 kb) [file 10460_2024_10651_MOESM1_ESM.docx]

| Name | Articles |
| --- | --- |
| Meat is harmful to planetary health | 59 |
| Us vs. them (outgroup polarisation) | 50 |
| UK livestock farming is efficient and sustainable | 38 |
| Livestock farming is part of the solution to climate change | 37 |
| Radical restraint is necessary (dramatic reduction in meat consumption) | 36 |
| DCD: Individualism | 30 |
| DCD: Whataboutism | 30 |
| Systemic solutions are needed to address climate impacts of ruminants | 30 |
| International trade and trends (influence emissions, including from ruminant livestock) | 27 |
| Imported foods are worse for the environment (than foods from ruminant livestock) | 23 |
| The media is biased and spreading disinformation | 23 |
| Politicians are not willing act on meat reduction | 20 |
| DCD: Technological optimism | 17 |
| Meat is beneficial to human health | 15 |
| Traditionality of grassland narrative (physical and human-made limitations of land) (Broomfield & Maher, 2022) | 15 |
| Climate campaigners are hypocrites | 14 |
| Grassland and hedges are important carbon sinks | 14 |
| Methane reduction is an opportunity to mitigate climate change | 14 |
| Agroecology and regenerative agriculture are sustainable approaches | 12 |
| Meat is harmful to human health | 12 |
| We need a more equitable food system | 11 |
| Meat is important for maintaining traditional UK landscapes | 10 |
| Ruminants are not a problem for the environment | 10 |
| The public need to be educated about methane emissions from ruminants | 10 |
| Proposed government action on methane emissions is suspicious and misguided | 9 |
| The livestock sector needs to speak out more | 9 |
| Vegan activists are a problem | 9 |
| DCD: All talk, little action | 8 |
| Environmentalists are guilty of green neocolonialism | 8 |
| Methane from ruminants is part of a cycle and therefore not a problem | 8 |
| The issue of methane emissions from ruminants is complex and nuanced | 8 |
| We should eat less and better meat | 8 |
| Appeal to nature (ruminants are natural) | 7 |
| Brazilian farmers and ranchers are responsible for the Amazon fires | 7 |
| GWP100 over-emphasises methane’s warming potential | 7 |
| The market will solve the problem of methane emissions from ruminants | 7 |
| Farmers' mental health is negatively impacted by these debates | 6 |
| DCD: Appeal to social justice | 5 |
| Food security is at risk in the UK | 5 |
| Pigs and poultry are less environmentally friendly than ruminants | 5 |

Supplementary Table 1: List of all discourses appearing in five or more articles

DCD: Discourse of Climate Delay

Bibliography

Broomfield, C. Y., & Maher, A. (2022). What role for England’s grassland farming regions in the transition to a sustainable food system? In D. Bruce & A. Bruce (Eds.), *Transforming food systems: ethics, innovation and responsibility* (pp. 180–185). Wageningen Academic Publishers. https://doi.org/10.3920/978-90-8686-939-8_26
